# Capsule Influences the Deposition of Critical Complement C3 Levels Required for the Killing of *Burkholderia pseudomallei* via NADPH-Oxidase Induction by Human Neutrophils

**DOI:** 10.1371/journal.pone.0052276

**Published:** 2012-12-14

**Authors:** Michael E. Woodman, Randall G. Worth, R. Mark Wooten

**Affiliations:** Department of Medical Microbiology and Immunology, University of Toledo College of Medicine, Toledo, Ohio, United States of America; Tulane University School of Medicine, United States of America

## Abstract

*Burkholderia pseudomallei* is the causative agent of melioidosis and is a major mediator of sepsis in its endemic areas. Because of the low LD_50_ via aerosols and resistance to multiple antibiotics, it is considered a Tier 1 select agent by the CDC and APHIS. *B. pseudomallei* is an encapsulated bacterium that can infect, multiply, and persist within a variety of host cell types. *In vivo* studies suggest that macrophages and neutrophils are important for controlling *B. pseudomallei* infections, however few details are known regarding how neutrophils respond to these bacteria. Our goal is to describe the capacity of human neutrophils to control highly virulent *B. pseudomallei* compared to the relatively avirulent, acapsular *B. thailandensis* using *in vitro* analyses. *B. thailandensis* was more readily phagocytosed than *B. pseudomallei*, but both displayed similar rates of persistence within neutrophils, indicating they possess similar inherent abilities to escape neutrophil clearance. Serum opsonization studies showed that both were resistant to direct killing by complement, although *B. thailandensis* acquired significantly more C3 on its surface than *B. pseudomallei*, whose polysaccharide capsule significantly decreased the levels of complement deposition on the bacterial surface. Both *Burkholderia* species showed significantly enhanced uptake and killing by neutrophils after critical levels of C3 were deposited. Serum-opsonized *Burkholderia* induced a significant respiratory burst by neutrophils compared to unopsonized bacteria, and neutrophil killing was prevented by inhibiting NADPH-oxidase. In summary, neutrophils can efficiently kill *B. pseudomallei* and *B. thailandensis* that possess a critical threshold of complement deposition, and the relative differences in their ability to resist surface opsonization may contribute to the distinct virulence phenotypes observed *in vivo*.

## Introduction

The causative agent of melioidosis, *Burkholderia pseudomallei*, is a saprophytic bacterium that is endemic throughout Southeast Asia and northern Australia [1], [2], [3]. This organism is a leading cause of pneumonia and septicemia in endemic areas [1], [2], [4], [5], [6]. In the Western hemisphere, *B. pseudomallei* have been documented sporadically in northern South America, Central America, and certain Caribbean islands, including Puerto Rico [7], [8], [9], [10], [11], [12], [13], and melioidosis cases are becoming increasingly more widespread in these and other tropical/sub-tropical areas worldwide [14], [15]. While infection can be established in healthy individuals through skin abrasions, ingestion, or inhalation, the incidence of melioidosis is more common in individuals with certain predisposing conditions, the primary one being diabetes mellitus [4], [16], [17]. Infection with *B. pseudomallei* can produce widely varying clinical symptoms which often confounds accurate diagnosis. Acute melioidosis is a serious condition that can rapidly become fatal, and is commonly characterized by abscess formation in lungs, liver, and/or spleen, as well as bacteremia. Latent melioidosis is characterized by a persistent infection that can recrudesce at varying times after the initial infection to cause disease, with the longest confirmed report being 62 years post-infection [18]. Notably, *B. pseudomallei* are extremely virulent via aerosol exposure, with an estimated LD_50_ between 5–100 organisms depending on the model [19], [20], [21]. Because of these characteristics, *B. pseudomallei* has recently been elevated to Tier 1 status by the CDC and APHIS [22]. *B. pseudomallei* is inherently resistant to many classes of antibiotics, and even treatment with proven antibiotics is often unsuccessful, with mortality rates for acute melioidosis ranging from 40–90% [2], [4]. No vaccine is currently available for preventing melioidosis, and there is great interest in identifying immune mechanisms that can promote efficient clearance of these infections.

While *B. pseudomallei* can be readily isolated as a free-living organism in moist tropical environments, it is also particularly efficient at infecting and persisting within both non-phagocytic and phagocytic host cell types. While not extensively studied, a number of potential virulence factors have been identified that may enhance their ability to persist intracellularly. These include type III and VI secretion systems which promote cell entry and rapid escape from endosomal compartments, as well as actin-based motility which allows for intercellular spread between adjacent cells without exposure to the extracellular milieu [23], [24], [25], [26], [27], [28], [29]. Capsule production is also known to be important for persistence in animal models of infection, although the specific virulence properties it provides is not well-established [30], [31]. One tool used to address the importance of putative virulence mechanisms are comparative studies using the closely-related, but relatively avirulent *B. thailandensis*. This sequenced bacterium does not produce the type I mannoheptose polysaccharide capsule expressed by *B. pseudomallei*, as well as lacks the ability to assimilate arabinose and a few additional genes for which no known virulence properties have been described [32], [33], [34], [35]. However, *B. thailandensis* does display an ability to escape the endosome, replicate, and persist in the cytoplasm in certain cell types *in vitro* [36], [37], [38]. While we still do not have a complete understanding of *B. pseudomallei* virulence mechanisms, it is evident that these bacteria are well-adapted to survive and persist within host cells, but our knowledge of which immune cells are critical for protection is limited.

Historically, the interaction between *B. pseudomallei* and macrophages has been a primary research focus, as macrophages are believed to be a major reservoir for both the replication and dissemination of these bacteria as well as for controlling these infections (reviewed in [39], [40]). However, recent i*n vivo* findings suggest neutrophils may also play a critical role in controlling *B. pseudomallei* infection, including the following: i. selective depletion of neutrophils in a mouse model leads to enhanced susceptibility to fatal melioidosis [41], ii. neutrophils are recruited to and interact with *B. pseudomallei* in infected lung tissues [41], [42], iii. mice lacking NADPH oxidase, an important enzyme in the generation of the microbicidal respiratory burst primarily utilized by neutrophils, are more susceptible to *B. pseudomallei* infection [43], iv. diabetes mellitus, which is the primary predisposing condition for melioidosis, is associated with impaired neutrophil function [44], [45], [46], [47], v. neutropenic individuals are more susceptible to *B. pseudomallei* infection and development of fatal disease [48], [49] and, vi. granulocyte colony-stimulating factor (G-CSF), which stimulates neutrophil differentiation, prolongs the survival of melioidosis patients, though a direct link to enhanced neutrophil function has not been proven [50], [51], [52], [53], [54].

Although *in vivo* studies suggest that neutrophils are important for controlling *B. pseudomallei* infection, a limited number of *in vitro* studies have provided conflicting findings on the ability of these phagocytes to directly clear *B. pseudomallei* [45], [55], [56], [57]. These reports have all varied as to neutrophil efficiency in phagocytosing and killing *B. pseudomallei*, their abilities to elicit an oxidative burst, and whether serum components provide any opsonizing properties for enhancing bacterial killing. Our current goal is to determine the ability of human primary neutrophils to clear the highly virulent *B. pseudomallei* compared to the relatively avirulent *B. thailandensis*, as well as delineate the mechanism(s) important for bacterial killing. Our findings are the first to demonstrate that neutrophils can effectively kill both *B. pseudomallei* and *B. thailandensis in vitro*, but only if sufficient complement deposition has occurred on the bacterial surface to activate an appropriate respiratory burst.

## Materials and Methods

### Bacterial culture and preparation

*B. pseudomallei* 1026b [58], *B. pseudomallei* DD503 [59], and *B. thailandensis* E264 [34] were a gift from Don Woods (University of Calgary). *B. pseudomallei* DD503 ΔLPS (BP2683) [60] and *B. pseudomallei* DD503 ΔCPS (SZ210) [30] were provided by Paul Brett and Mary Burtnick (University of Southern Alabama). *Escherichia coli* strain K12 substrain W3110 was used as a control in specific experiments. For these studies, all bacterial strains were cultured aerobically for 18 hours at 37°C on tryptic soy agar (TSA) (Neogen) plates. Bacteria were recovered by scraping from TSA plates into phosphate buffered saline (PBS) and initially enumerated using a spectrophotometer, which was confirmed by dilution plating. All studies utilizing live *B. pseudomallei* were conducted in a CDC select agent-certified BSL3 laboratory.

### Serum opsonization of bacteria

For experiments involving serum opsonization, bacteria (1×10^8^ CFU) were incubated with the described concentrations of pooled normal human serum (NHS) (Complement Technology) or heat-inactivated (HI) serum in PBS containing 0.25 µM CaCl_2_ and 1 µM MgCl_2_ at 37°C for 30 min. HI serum was prepared by incubating the pooled human serum at 56°C for 30 min prior to addition to bacteria. To evaluate the relative contributions of the classical, lectin and alternative complement pathways to bacterial opsonization, either 10 mM ethylenediaminetetraacetic acid (EDTA) (blocks classical, lectin and alternative pathways by chelating both calcium and magnesium) or a combination of 5 mM magnesium chloride and 5 mM ethylene glycol tetraacetic acid (MgEGTA) (only blocks classical and lectin pathways by preferentially chelating calcium over magnesium) final were added, respectively.

### Quantification of complement deposition and antibody binding on bacterial surfaces

All bacteria were opsonized with NHS as described above. Opsonized bacteria were then washed with PBS to remove any unbound components and fixed with 1% paraformaldehyde. Fixed bacteria were washed and labeled with goat anti-human C3 polyclonal IgG-FITC conjugated (MP Biomedicals) at 1∶400, washed, and analyzed by flow cytometry using a BD FACSCalibur (Becton Dickinson). To assess the presence of bacteria-specific antibodies, bacteria opsonized with NHS were subsequently incubated with both APC-labeled donkey anti-human IgM (1∶100) and R-PE-labeled donkey anti-human IgG (1∶100) (Jackson ImmunoResearch), washed, and analyzed by flow cytometry. Results are reported as mean fluorescence intensity (MFI).

### Serum-mediated killing of bacteria

All *B. pseudomallei* strains, *B. thailandensis*, and *E. coli* (serum-sensitive) were incubated with 0, 20, 40 or 80% NHS and 40% HI at a concentration of 10^6^ bacteria/ml under the same conditions as described for bacterial opsonization assays. At 0, 2, and 4 h post-incubation, an aliquot of each sample was serially diluted and plated on TSA to determine CFU/ml.

### Neutrophil isolation from whole human blood

All studies involving human samples were in accordance with and approved by the University of Toledo Biomedical Institutional Review Board (IRB). Neutrophils were isolated from whole venous blood obtained from healthy human volunteers, as previously described [61]. Briefly, heparinized blood was combined with equal parts 3% dextran at room temperature for 20 min to sediment erythrocytes. The leukocyte suspension was centrifuged at 500×g for 10 min, the cells were resuspended in PBS and underlayed with equal volumes Ficoll-sodium metrizoate solution (density 1.077 g/ml) (MP Biomedicals), and centrifuged for 40 min at 400×g at room temperature with no brake. The erythrocyte/granulocyte-rich pellet was resuspended in sterile water for 25 s to allow hypotonic lysis of erythrocytes, and tonicity was restored by addition of PBS. The remaining granulocytes were >95% neutrophils and >95% cell viability as determined by Wright-Giemsa and trypan blue staining, respectively.

### Bacterial killing by neutrophils

Human neutrophils were seeded at 1×10^6^ per well in 24 well plates containing 0.5 ml RPMI 1640 with HL-1 supplement (Lonza) and Glutamax (Invitrogen)(i.e. “complete RPMI”). Neutrophils were incubated at 37°C in 5% CO_2_ for 20 min before addition of *B. pseudomallei* or *B. thailandensis* at an MOI = 1. In specific experiments, diphenyleneiodonium (DPI; a NADPH-oxidase inhibitor) (10 µM final) or a DMSO vehicle control was added during this incubation. The plates were centrifuged at 250×*g* for 5 min to synchronize infection and allowed to incubate at 37°C in 5% CO_2_ for 10 min before washing 3x with RPMI to remove extracellular bacteria. No antibiotics were used to kill any remaining extracellular bacteria because they have been demonstrated to enter phagocytes and kill intracellular bacteria [62], [63], [64], [65]. The neutrophils in these parallel co-cultures were lysed at either 10 min (i.e. to assess uptake) or 2 h after infection (i.e. to assess clearance) with a 0.5% saponin solution, and intracellular bacteria were enumerated by serial dilution on TSA.

### Quantification of neutrophil respiratory burst

Human neutrophils were seeded at 2×10^5^ per well in 96 well plates in PBS containing calcium and magnesium in the presence or absence of DPI (10 µM final). Neutrophils were pretreated with luminol (50 µM 3-aminophthalhydrazide in 0.1 M NaOH) at 37°C in 5% CO_2_ for 20 min before addition of *B. pseudomallei* or *B. thailandensis* at an MOI = 1. Co-cultures were centrifuged at 500×*g* for 1 min to initiate bacteria:neutrophil contact and immediately analyzed on a FLUOstar Omega plate reader (BMG Labtech), with luminescence detection every min for 20 min.

### Statistical analysis

Graphpad Instat (La Jolla, CA) was used for all statistical analyses. Statistical differences were determined by either performing a two-way T-test or one-way ANOVA followed by a Tukey's post-hoc test (*P*≤0.05).

## Results

### Phagocytosis and clearance of unopsonized *Burkholderia* species by human neutrophils

Neutrophils are observed to be rapidly recruited to the site of infection during experimental respiratory melioidosis and blocking this influx results in greater *B. pseudomallei* numbers and mortality [41], [42]. However, previous *in vitro* studies have been conflicted on the ability of neutrophils to directly clear *B. pseudomallei* [45], [55], [56], [57]. We sought to clarify this by evaluating the ability of and mechanism(s) necessary for human neutrophils to phagocytose and kill highly virulent *B. pseudomallei*. We also sought to determine if there was differential killing of *B. pseudomallei* by neutrophils compared to that of the closely-related, but relatively avirulent *B. thailandensis*.

**Figure 1:**
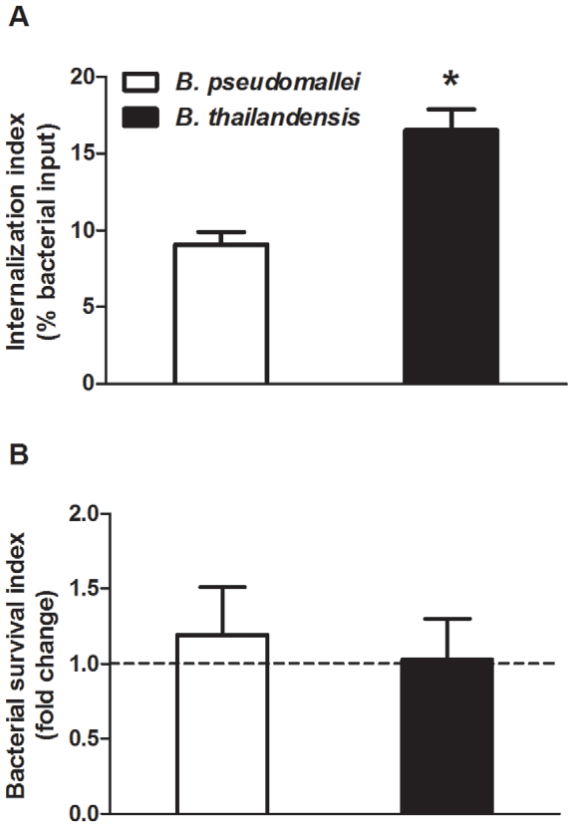
*B. pseudomallei* and *B. thailandensis* are inherently resistant to killing by human neutrophils. (A) *B. pseudomallei* and *B. thailandensis* were either incubated with neutrophils for 10 min to measure bacterial uptake (% bacterial input) or (B) for 2 h to measure bacterial survival (fold change from respective uptake values) as described in the Materials and Methods. The bars represent the mean±SEM of three separate experiments using neutrophils from different donors, each performed in duplicate. * indicates a statistically significant difference (*P*≤0.05) between *B. pseudomallei* and *B. thailandensis* values.

The inherent capability of neutrophils to internalize and clear *B. pseudomallei* and *B. thailandensis* was assessed in Figure 1. Human neutrophils were able to internalize both unopsonized *B. pseudomallei* and *B. thailandensis*, although the uptake of *B. thailandensis* (16.5%) by neutrophils was significantly greater than *B. pseudomallei* (9.1%) (Figure 1A). Subsequently, there was no change in bacterial viability of either species after 2 h incubation with neutrophils compared to the initial uptake (Figure 1B). Thus, though human neutrophils inherently internalize *B. thailandensis* more efficiently than *B. pseudomallei*, they are subsequently unable to clear either bacterial species.

### Complement deposition on the surface of *B. pseudomallei* and *B. thailandensis*

**Figure 2:**
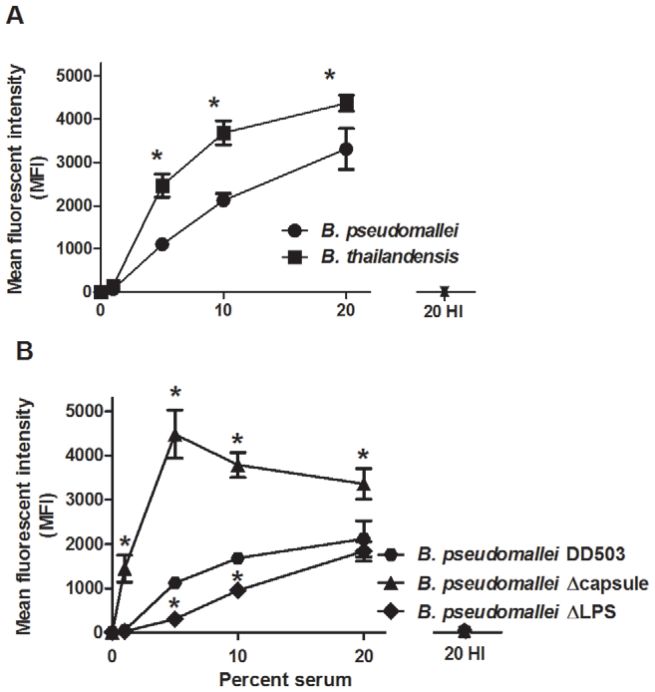
*B. pseudomallei* acquire less complement C3 on its surface than *B. thailandensis.* (A) *B. pseudomallei* 1026b and *B. thailandensis* E264, or (B) *B. pseudomallei* DD503, *B. pseudomallei* ΔCPS, and *B. pseudomallei* ΔLPS were opsonized for 30 min at the indicated serum concentrations, washed, and stained with an anti-human C3 FITC-conjugated antibody. Samples were analyzed by flow cytometry and the data reported as mean fluorescence intensity (MFI). The bars represent the mean±SEM of three separate experiments performed with duplicate samples. (A) * indicates a statistically significant difference (*P*≤0.05) between *B. pseudomallei* and *B. thailandensis* values; ns  =  not significant. (B) * indicates a statistically significant difference (*P*≤0.05) compared to *B. pseudomallei* DD503 (wild-type).

Before assessing the effects of serum opsonization on neutrophil responses to *B. pseudomallei* or *B. thailandensis*, the relative levels of complement deposition on the surface of these bacteria were measured in the presence of different concentrations of normal human serum (NHS), using flow cytometry (Figure 2A). After incubation with 5%, 10% and 20% NHS, significant levels of complement component C3 were detected on the surface of both *Burkholderia* species compared to unopsonized bacteria, whereas 1% NHS opsonization did not promote significant C3 deposition. In general, increased serum concentrations correlated with increased C3 deposition in both species. However, *B. thailandensis* did acquire significantly greater levels of C3 deposition at 5%, 10% and 20% NHS compared to *B. pseudomallei*. Bacteria incubated with heat-inactivated (HI) serum did not acquire substantial C3 on their surfaces. These data indicate that *B. pseudomallei* are more resistant to C3 deposition compared to *B. thailandensis*.

Two *B. pseudomallei* factors reported to be involved in serum resistance are the polysaccharide capsule and lipopolysaccharide (LPS), respectively [30], [66]. To determine if either of these factors may explain the increased C3 deposition on *B. thailandensis* compared to *B. pseudomallei*, similar experiments were performed using both capsule-deficient (ΔCPS) and LPS-deficient (ΔLPS) *B. pseudomallei* mutants (Figure 2B). The ΔCPS mutant showed significantly increased C3 deposition on its surface compared to the wild-type control (DD503) at all serum concentrations measured. The ΔLPS mutant showed the opposite trend, with C3 deposition being significantly decreased compared to the control at 5% and 10% NHS. These data suggest that the *B. pseudomallei* capsule is responsible for the differences in C3 deposition observed between *B. pseudomallei* and *B. thailandensis*.

**Figure 3:**
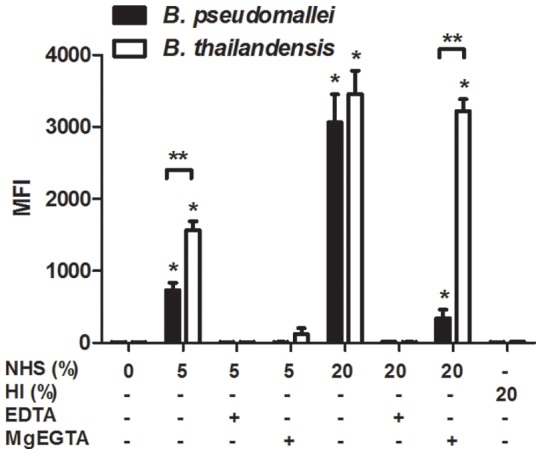
*B. pseudomallei* are resistant to C3 deposition by the alternative pathway of complement. *B. pseudomallei* and *B. thailandensis* were incubated with the indicated serum concentrations in the presence or absence of MgEGTA or EDTA, washed, and stained with an anti-human C3 FITC-conjugated antibody. Samples were analyzed by flow cytometry and the data reported as mean fluorescence intensity (MFI). The bars represent the mean±SEM of three separate experiments performed with duplicate samples. The single asterisk (*) indicates a statistically significant increase (*P*≤0.05) over unopsonized bacteria. The double asterisks (**) indicate a statistically significant difference (*P*≤0.05) between *B. pseudomallei* and *B. thailandensis* values.

**Figure 4:**
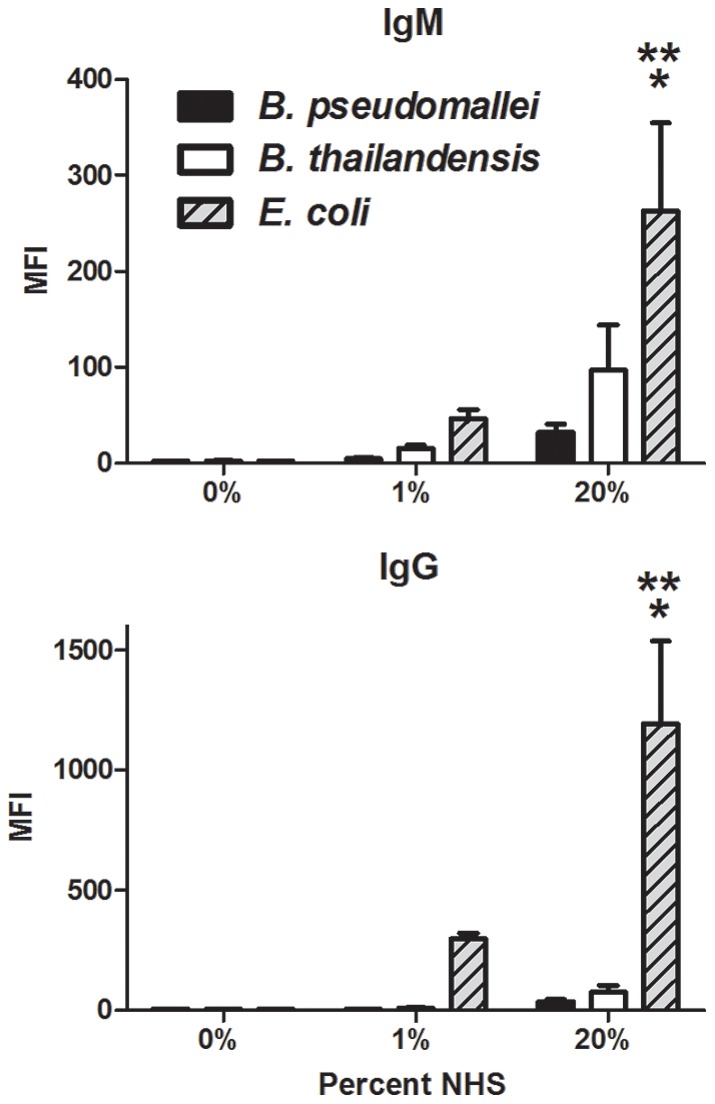
Levels of endogenous *Burkholderia*-reactive antibodies present in normal human serum. *B. pseudomallei,* B. *thailandensis*, and *E. coli* were incubated with the indicated serum concentrations, washed, and stained with both an APC-labeled anti-human IgM antibody and R-PE-labeled anti-human IgG antibody. Samples were analyzed by flow cytometry and the data reported as mean fluorescence intensity (MFI). The bars represent the mean±SEM of two separate experiments performed with duplicate samples. The single asterisk (*) indicates a statistically significant increase (*P*≤0.05) over unopsonized bacteria. The double asterisks (**) indicate a statistically significant difference (*P*≤0.05) over 20% NHS-opsonized *B. pseudomallei* and *B. thailandensis* values.

To address which activation pathway(s) were responsible for complement deposition on *B. pseudomallei* and *B. thailandensis*, both bacterial species were incubated with 5% and 20% NHS in the presence or absence of EDTA (prevents activation of all complement pathways) or MgEGTA (allows only alternative pathway activation), and C3 deposition analyzed by flow cytometry (Figure 3). Addition of EDTA to NHS-opsonized samples reduced C3 deposition levels to that of unopsonized bacteria for both *B. pseudomallei* and *B. thailandensis*, as expected. MgEGTA addition to the 5% NHS samples allowed minimal C3 surface deposition on both bacteria, suggesting that complement deposition at 5% NHS is largely dependent on the classical or lectin pathways and that both are resistant to alternative pathway-activation at that serum concentration. After incubation in 20% NHS with MgEGTA, C3 binding to *B. pseudomallei* was significantly reduced compared to in the absence of MgEGTA. This decrease in C3 deposition was not observed for *B. thailandensis* opsonized in 20% NHS and MgEGTA, demonstrating efficient alternative pathway activation. These results suggest that *B. pseudomallei* are resistant to alternative pathway activation even at high serum concentrations, whereas *B. thailandensis* is highly susceptible at these serum levels. Surprisingly, the substantial complement deposition that occurs in NHS for both *Burkholderia* species appears largely due to the classical or lectin pathway. To attempt to differentiate between these pathways, we measured the levels of IgG and IgM in NHS that could bind *B. pseudomallei* and *B. thailandensis* by flow cytometry. The endogenous levels of *B. pseudomallei* and *B. thailandensis-*reactive antibodies in NHS were not significantly different from unopsonized bacteria, whereas IgG and IgM specific for a commensal bacterium (i.e. *E. coli*) were significantly elevated in 20% NHS (Figure 4). It is currently unclear if these very low endogenous levels of *Burkholderia*-reactive IgG or IgM in NHS are enough to activate the classical pathway versus the contribution of innate immune mediators that activate via the classical or lectin pathway.

### Resistance of *Burkholderia* species to direct killing by serum

**Figure 5:**
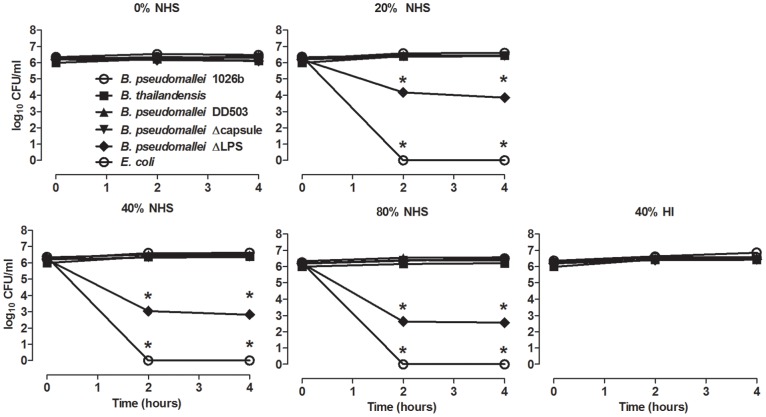
*B. pseudomallei* and *B. thailandensis* are resistant to direct complement-mediated killing. *B. pseudomallei* 1026b, *B. thailandensis, B. pseudomallei* DD503, *B. pseudomallei* ΔCPS, *B. pseudomallei* ΔLPS and *E. coli* were incubated at the indicated serum concentrations at 37°C. At the indicated times, an aliquot of each sample was serially diluted and plated on TSA to determine CFU/ml. Each condition was assessed as duplicate samples in all experiments. * indicates a significant difference (*P*≤0.05) between *E. coli* and both *Burkholderia* species.

To determine whether the observed differences in surface C3 deposition might directly affect survival of *B. pseudomallei* or *B. thailandensis*, the bacteria were incubated in NHS and enumerated at different times post-incubation to assess the direct killing effect. *B. pseudomallei* viability was unaltered up to 4 h post-incubation in 20%, 40%, and 80% NHS, confirming it is resistant to serum bactericidal activity (Figure 5) [30], [55], [66], [67]. Although, *B. thailandensis* was shown to have much higher levels of C3 deposition (Figure 2), it demonstrated similar resistance to direct killing as *B. pseudomallei* at all serum concentrations, as has been previously suggested [66]. The ΔCPS *B. pseudomallei* was as serum resistant as wild-type *B. pseudomallei* and *B. thailandensis*, indicating the capsule is not required for survival in serum. However, the ΔLPS *B. pseudomallei* showed significant death in 20, 40 and 80% serum, even though it had very low levels of C3 deposition (Figure 2). The results suggest that LPS attracts C3 deposition, but does not allow MAC formation capable of disrupting the bacterial outer membrane. As a control, no viable *E. coli* (serum-sensitive) were detected after a 2 h incubation in ≥20% NHS, demonstrating the NHS possessed bactericidal activity. Direct killing of *E. coli* was not observed in 40% HI serum, indicating the mechanism was complement-mediated. These findings suggest that, even though there are significant differences in surface C3 deposition between *B. pseudomallei* and *B. thailandensis*, both possess a similar resistance to direct killing by complement. Additionally, while LPS seems to be necessary for the survival of *B. pseudomallei* in serum, the capsule is not involved in resistance to serum-mediated direct killing.

### Serum opsonization of *B. pseudomallei* and *B. thailandensis* results in increased uptake and killing by human neutrophils

**Figure 6:**
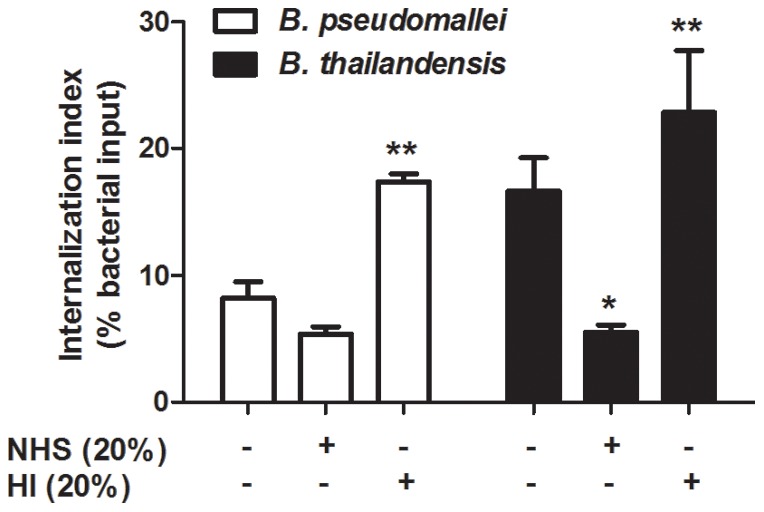
Effects of serum opsonization on *B. pseudomallei* and *B. thailandensis* uptake by neutrophils. Bacteria were incubated in the presence or absence of the indicated sera prior to co-culture with neutrophils. Bacterial uptake was measured at 10 min post-infection. The bars represent the mean±SEM of three separate experiments using neutrophils from different donors and performed in duplicate. The single asterisk (*) indicates a significant difference (*P*≤0.05) between unopsonized and 20% NHS opsonized *B. thailandensis* values. The double asterisks (**) indicate a statistically significant difference (*P*≤0.05) between 20% NHS and 20% HI opsonized bacteria.

Although neutrophils could not kill unopsonized *B. pseudomallei* or *B. thailandensis*, it is possible that surface C3 deposition could promote phagocytosis and/or killing by neutrophils. Initial studies measuring bacterial uptake by neutrophils at 10 min post-infection showed equal or decreased numbers of internalized serum-opsonized *B. pseudomallei* and *B. thailandensis*, respectively, compared to unopsonized bacteria (Figure 6). When the bacteria were opsonized with HI serum, the number of internalized *B. pseudomallei* and *B. thailandensis* appeared to increase over that of their respective unopsonized numbers. However, a significant decrease in uptake was observed between bacteria opsonized with 20% NHS compared to 20% HI for both species, indicating a role for a heat-labile serum factor(s), most likely a component of the complement cascade. However, these findings were counter-intuitive and were not consistent with any trends reported for similar intracellular bacteria (reviewed in [68], [69]).

**Figure 7:**
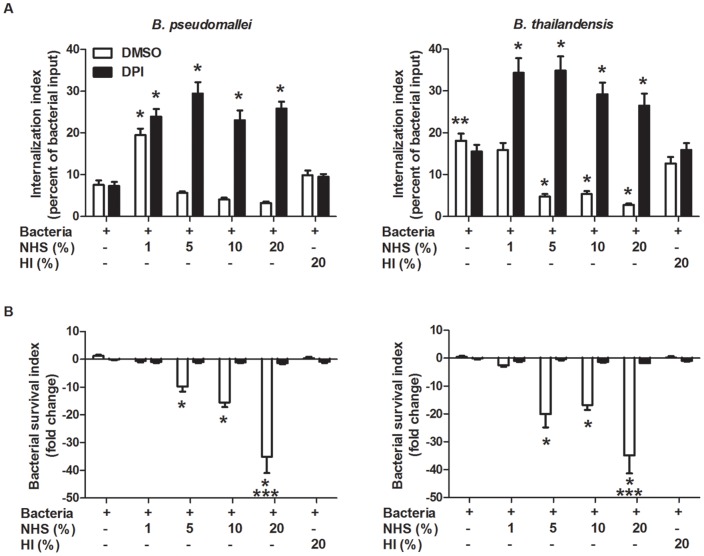
Serum opsonization enhances internalization and clearance of *B. pseudomallei* and *B. thailandensis* by neutrophils. Isolated human neutrophils were treated with DPI or DMSO (vehicle control) for 20 min prior to bacterial infection. Neutrophils were incubated with *B. pseudomallei* and *B. thailandensis* for (A) 10 min to measure bacterial uptake or for (B) 2 h to measure bacterial survival. The bars represent the mean±SEM of three separate experiments using neutrophils from different donors and performed in duplicate. The single asterisk (*) indicates a significant difference (*P*≤0.05) compared to unopsonized bacteria. The double asterisks (**) indicate a significant difference (*P*≤0.05) between *B. pseudomallei* and *B. thailandensis*. The triple asterisks (***) indicate a significant difference (*P*≤0.05) between 20% NHS opsonized *Burkholderia* species and all other conditions.

One possibility for these misleading results is that serum-opsonized *B. pseudomallei* and *B. thailandensis* were being rapidly killed by neutrophils, producing an artificially low internalization rate. To address this, the internalization studies were repeated in the presence or absence of diphenyleneiodonium (DPI), a NADPH-oxidase inhibitor that blocks the respiratory burst. Internalization of unopsonized *B. thailandensis* by neutrophils at 10 min post-infection was again significantly greater than for unopsonized *B. pseudomallei*, and this was unaltered by the addition of DPI (Figure 7A). *B. pseudomallei* opsonized in 1% NHS showed significantly greater internalization by neutrophils compared to unopsonized bacteria, both in the absence and presence of DPI. *B. thailandensis* opsonized with 1% NHS showed no change in internalization by neutrophils compared to unopsonized bacteria, but DPI-treated neutrophils had significantly more internalized *B. thailandensis*. Internalization of *B. pseudomallei* opsonized in 5%, 10%, and 20% NHS-opsonized *B. pseudomallei* was low in the absence of DPI; however, neutrophils treated with DPI showed significantly enhanced uptake of *B. pseudomallei* opsonized at the same serum concentrations. *B. thailandensis* opsonized with 5%, 10%, and 20% NHS were taken up at significantly lower numbers in the absence of DPI than unopsonized bacteria. However, in the presence of DPI, *B. thailandensis* opsonized at the same NHS concentrations showed significantly enhanced internalization by neutrophils, and these levels were similar to that seen for *B. pseudomallei*. Opsonization of both *B. pseudomallei* and *B. thailandensis* in 20% HI serum had no effect on internalization compared to unopsonized bacteria. These data suggest that serum opsonization increases the ability of neutrophils to internalize both *B. pseudomallei* and *B. thailandensis* in a complement-dependent manner. Additionally, bacterial opsonization in ≥5% serum elicits a rapid reduction in numbers of viable bacteria, which is associated with the respiratory burst (i.e. DPI-sensitive). Interestingly, *B. pseudomallei* opsonized with 1% NHS demonstrated increased internalization by neutrophils but did not stimulate bactericidal activity, whereas *B. thailandensis* showed increased uptake and rapid killing at this serum concentration, suggesting there are differences in the amount of reactive oxygen species (ROS) produced in response to these species.

The ability of *B. pseudomallei* and *B. thailandensis* to subsequently survive after internalization was measured in the absence and presence of DPI for 2 h post-infection. The numbers of unopsonized *B. pseudomallei* and *B. thailandensis* within neutrophils were not reduced during the 2 h assessment period (Figure 7B). Opsonization of *B. pseudomallei* and *B. thailandensis* in 1% serum produced some reduction in bacterial numbers, but these values were not significant. Opsonization of both species in 5% and 10% serum elicited a significant reduction in intracellular numbers, and increasing the serum concentration to 20% produced a further significant reduction in bacterial numbers compared to the lower serum concentrations. Notably, opsonization of both species in 20% HI serum produced no reduction in bacterial numbers, and neutrophils treated with DPI were unable to reduce the numbers of either *B. pseudomallei* or *B. thailandensis* regardless of the serum concentration used for opsonization. The results demonstrate that opsonization of both *B. pseudomallei* and *B. thailandensis* with ≥5% NHS elicits significant activation and bactericidal activity by human neutrophils, and this is dependent on induction of a respiratory burst.

### Bacterial opsonization with serum is required for rapid induction of the neutrophil respiratory burst

**Figure 8:**
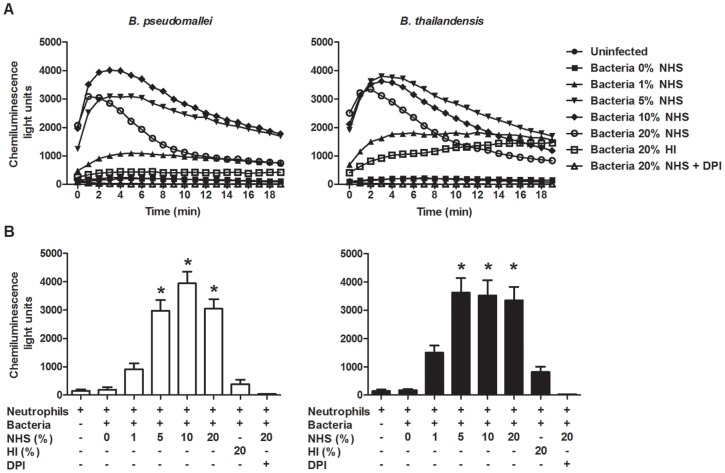
Correlation of serum-opsonization and induction of respiratory burst by *B. pseudomallei* and *B. thailandensis*. (A) Kinetics of reactive oxygen species produced by neutrophils following bacterial infection. Neutrophils were pre-incubated in the presence of luminol before addition of *B. pseudomallei* or *B. thailandensis* at an MOI = 1 with or without DPI. Co-cultures were centrifuged briefly to initiate bacteria:neutrophil contact and immediately analyzed on a plate reader with luminescence detection continuously for a total of 20 min. The data represents the combined results of three separate experiments using neutrophils from different donors and performed in triplicate. (B) Chemiluminescence levels measured at t = 2 min post-infection. The bars represent the mean±SEM of three separate experiments performed in triplicate. * indicates a statistically significant difference (*P*≤0.05) compared to neutrophils stimulated with unopsonized *B. pseudomallei* or *B. thailandensis*.

To further delineate the affects of serum opsonization on eliciting neutrophil killing, a luminol-based chemiluminescence assay was utilized to quantify the kinetics and magnitude of the neutrophil respiratory burst induced by *B. pseudomallei* and *B. thailandensis*. Neutrophils co-cultured with unopsonized bacteria did not induce a respiratory burst and appeared similar to uninfected cells (Figure 8A). Neutrophils co-cultured with *B. pseudomallei* and *B. thailandensis* opsonized in 5%, 10%, or 20% NHS induced a rapid and substantial respiratory burst compared to unopsonized bacteria, whereas bacteria opsonized in 1% serum produced a more intermediate response. The maximum respiratory burst for *B. pseudomallei* and *B. thailandensis* opsonized with ≥5% serum was reached within 2–3 min after inoculation. These values were all significantly greater than neutrophils exposed to unopsonized bacteria, which produced little to no ROS (Figure 8B). Neutrophils infected with either bacteria opsonized with 1% serum did show some increase in ROS activity, but these levels were not significantly different from background values. It is noteworthy that we also did not observe significant neutrophil killing of *B. pseudomallei* or *B. thailandensis* opsonized with 1% NHS (Figure 7B). Neutrophils infected with 20% HI opsonized *B. pseudomallei* or *B. thailandensis* produced baseline levels of ROS, reiterating the importance of complement deposition in inducing the respiratory burst against these *Burkholderia* species.

## Discussion

Melioidosis can be a highly lethal disease if not appropriately diagnosed and treated, particularly with the development of pneumonia and bacteremia that frequently leads to involvement of multiple organs. A major issue in controlling these infections is that *B. pseudomallei* are highly efficient at infecting and persisting within multiple non-immune and immune cell types. Within susceptible cell types, these bacteria can quickly escape endosomal compartments and subsequently utilize actin polymerization to efficiently invade adjacent cells without being exposed to the extracellular environment, thus limiting their exposure to antibodies and other soluble immune effectors. Therefore, it is important to identify immune cells involved in the cellular response that are best able to control these infections, as well as the mechanisms that promote bacterial clearance. Neutrophils are important for controlling systemic infections caused by numerous bacterial species, including many that are associated with pneumonia and bacteremia [70]. While neutrophils have been demonstrated to be critical for controlling melioidosis *in vivo* directly through depletion studies, it is still unclear if the neutrophils have a direct effect on *B. pseudomallei* clearance or the cells have an indirect role through modulation of other cell types [41]. A recent study conversely indicated that neutrophil recruitment during melioidosis may be detrimental in controlling bacterial numbers and host survival, and suggested monocytes may be important to limit *B. pseudomallei* infection [71]. There is indirect evidence that neutrophils may play a role in controlling melioidosis infection through correlative findings based on cellular recruitment to the infection site, predisposing conditions, and adjunctive therapies; however any protective properties have not been clearly confirmed to be attributable to neutrophils [44], [45], [46], [47], [48], [49], [50], [51], [52], [53], [54]. Notably, *in vitro* studies to delineate the relative abilities of neutrophils to kill *B. pseudomallei* have provided conflicting results [45], [55], [56], [57]. In our current study, we sought to determine the requirements that allow human neutrophils to kill *B. pseudomallei*, the mechanisms needed for this process, and whether there are differences in killing efficiencies between the relatively avirulent *B. thailandensis* and highly virulent *B. pseudomallei* that correlate with their contrasting pathogenesis *in vivo*.

When neutrophils were assessed for their inherent abilities to neutralize these bacteria, both species were recognized and phagocytosed within 10 min of co-incubation, although *B. thailandensis* was taken up at twice the rate as *B. pseudomallei*. Regardless, both *B. pseudomallei* and *B. thailandensis* showed similar abilities to subsequently resist killing and persist within neutrophils. One explanation for the differences in uptake could be the capsule produced by *B. pseudomallei* makes it more difficult for neutrophils to recognize and/or internalize compared to the acapsular *B. thailandensis*. Such anti-phagocytic properties have been described for a number of Gram-positive and Gram-negative bacteria possessing a carbohydrate capsule, including *Streptococcus pneumoniae, Staphylococcus aureus, Neisseria meningitis, Haemophilus influenzae, Klebsiella pneumoniae*, and *Escherichia coli* (reviewed in [72], [73], [74], [75]). However, possession of this capsule alone does not correlate with their ability to evade neutrophil clearance, as acapsular *B. thailandensis* displayed a similar ability to resist neutrophil killing as *B. pseudomallei*. Thus, both bacteria appear able to inherently evade neutrophil clearance and additional immune mechanisms must be involved if neutrophils are able to control these infections *in vivo*.

Antibodies and other serum opsonins are known to be crucial for neutrophils to recognize and kill certain bacteria, and particularly those that possess a capsule (reviewed in [72], [73], [74], [75]). We chose to focus on complement as a critical opsonin to promote efficient killing of these *Burkholderia* species, particularly since these innate components would be present early during infection and before the development of *Burkholderia*-specific antibodies. Quantification of C3 deposition on the bacterial surface indicated that *B. thailandensis* acquired significantly more C3 on its surface compared to *B. pseudomallei*. Parallel studies using the ΔCPS *B. pseudomallei* mutant suggested that this capsular material is largely responsible for the reduced C3 levels deposited on *B. pseudomallei*, and titration studies indicated that this protection was most apparent in low levels of serum/complement, which likely reflects the levels encountered in most host tissues. When serum levels were relatively low (≤5%), components of the classical or lectin pathways were necessary for complement activation on both bacterial species; however when the serum concentration was increased to 20% NHS, activation through the alternative pathway contributed to the majority of C3 on *B. thailandensis*, but not for *B. pseudomallei.* Although, *B. thailandensis* acquired more surface C3 than *B. pseudomallei*, both *Burkholderia* species were equally resistant to complement-mediated direct killing. The mechanism(s) that *Burkholderia* species use to resist direct killing by complement is not known. While *B. pseudomallei* LPS is known to be involved in serum resistance [66], and our findings indicate it is absolutely required for the complete complement resistance observed by *B. pseudomallei*, it has not been determined how it mediates this effect and what host or other bacterial factors are involved in this process. The length of the LPS O-antigen has been associated with serum resistance in some Gram-negative bacteria [76], and the structure of *B. pseudomallei* and *B. thailandensis* O-antigen are similar, suggesting this could represent a common serum-resistance mechanism between these closely-related bacterial species [34], [77], [78]. Since the *B. pseudomallei* ΔLPS mutant had less C3 deposition than wild-type *B. pseudomallei* but still resulted in direct bacterial killing, this indicated that C3 is directly deposited onto LPS and may thus prevent the assembly of the MAC on the bacterial outer membrane. Multiple bacterial species including *Haemophilus influenzae*, *Neisseria meningitidis* and *N. gonorrhoeae, Borrelia burgdorferi, Streptococcus pyogenes*, and *Moraxella catarrhalis* bind negative regulators of the complement system as a means to avoid direct killing by complement (reviewed in [79]), particularly via the alternative pathway. However, there have been no reports that *B. pseudomallei* or *B. thailandensis* can similarly bind complement regulatory proteins to avoid direct killing. Our data also indicate that *B. pseudomallei* are resistant to activation of the complement system by the alternative pathway. This finding goes against previous studies showing the alternative pathway is the predominant pathway of complement activation on *B. pseudomallei* [55], [66]. This discrepancy may be explained by differences in serum concentration and incubation times between these studies. While our studies clearly show that activation via the classical or lectin pathway is initiated in response to *B. pseudomallei* and *B. thailandensis* in NHS, it is still unclear which molecule(s) present in NHS is initiating these pathways for both *Burkholderia* species, and future studies will address this finding. There is evidence that NHS contains both IgG and IgM “natural” antibodies that can inherently bind to many bacterial surfaces and activate the classical pathway of complement [80], [81], although the levels of *Burkholderia*-specific antibodies we detected in NHS for our studies was extremely low (Figure 4). Alternatively, the pentraxins serum amyloid P (SAP) and C-reactive protein (CRP) are known to bind certain sugars present on the surfaces of bacteria to directly activate the classical pathway, and CRP can also interact with ficolin to activate the lectin-mediated pathway [82], [83]. The identification of the innate mediators responsible for this complement activation may provide innovative targets for *Burkholderia*-targeted innate immunotherapies.

Because of the significant differences in surface C3 deposition observed between *B. pseudomallei* and *B. thailandensis*, particularly at lower serum concentrations, we hypothesized that neutrophils would internalize and kill opsonized *B. thailandensis* more effectively than *B. pseudomallei*. Serum opsonization did elicit a significant increase in phagocytosis and killing of both *Burkholderia* species by neutrophils, and these activities did correlate with the relative levels of C3 surface deposition on these bacteria. *B. pseudomallei* and *B. thailandensis* opsonized with ≥5% NHS were both internalized more efficiently than unopsonized bacteria, and subsequently were cleared at a significantly greater rate within 2 h of co-culture. Increasing the NHS levels to 20% did not change the uptake rate, but did significantly enhance the clearance levels of both strains over the lower serum concentrations. Interestingly, when *B. pseudomallei* were opsonized with 1% NHS, an increased uptake by neutrophils was observed both in the presence and absence of the NADPH-oxidase inhibitor, whereas 1% serum-opsonized *B. thailandensis* only showed increased uptake in the presence of the inhibitor. These results suggest that the different degrees of C3 deposition observed in 1% serum represent the threshold levels required for enhanced bacterial uptake (observed in both strains opsonized in 1% NHS) versus the levels required to initiate the respiratory burst; i.e. the uptake levels for *B. pseudomallei* opsonized in 1% NHS were not increased in the presence of DPI. This suggests the C3 levels on *B. pseudomallei* were not sufficient to initiate the ROS production that was observed for *B. thailandensis* in 1% NHS, where the enhanced uptake could only be visualized after adding the NADPH-oxidase inhibitor. These observations also correlate with our direct *in vitro* analyses demonstrating that certain serum levels are required to elicit neutrophil NADPH oxidase activity to clear *B. pseudomallei*. Bacteria opsonized in ≥5% NHS induce significant ROS generation by neutrophils compared to unopsonized bacteria, with maximum levels acquired within 2–4 minutes of bacterial contact. The rapidity of the ROS generation is likely critical for *B. pseudomallei* clearance, as these bacteria are known to escape from phagosomal/endosomal compartments within 15–20 minutes of uptake [26], [84]. Bacteria opsonized in 1% NHS elicited a greatly reduced ROS response, which corresponds to the inefficient clearance observed for these bacteria. Interestingly, bacteria opsonized with 20% NHS elicited similar ROS levels as bacteria opsonized in 5% and 10% NHS (Figure 8), however the 20% opsonized bacteria were cleared at a significantly higher rate than the 5–10% opsonized bacteria (Figure 7). This suggests that other neutrophil-related mechanisms besides the respiratory burst may become activated in response to these higher levels of C3 deposition and contribute to killing of these *Burkholderia* species *B. pseudomallei* are susceptible to the bactericidal action of certain antimicrobial peptides that are produced by neutrophils, specifically the cathelicidin peptide, LL-37 [85], [86], [87]. In addition, neutrophils contain a wide variety of antimicrobial molecules including additional antimicrobial peptides or defensins, myeloperoxidase, neutrophil extracellular traps (NETs), and serine proteases which could work in concert with ROS generation [88], [89], [90], [91], [92], [93]. NETs were recently demonstrated to be antibacterial against *B. pseudomallei*, and NET release in response to *B. pseudomallei* was NADPH-oxidase dependent, as has been demonstrated previously with other agonists [94], [95], [96]. Since those studies were performed with unopsonized *B. pseudomallei*, our findings indicate that NET release may occur at a much faster rate with serum-opsonized bacteria in coordination with the rapid induction of the respiratory burst. Altogether, our data demonstrate a clear requirement for complement opsonization to allow for efficient uptake and killing of *B. pseudomallei* by neutrophils, and this bacterial clearance is dependent on achieving a rapid respiratory burst that elicits a threshold level of ROS generation.

Although melioidosis could historically be considered a neglected tropical disease, the global interest in these infections has increased dramatically in the past decade. The regions where *B. pseudomallei* has been recovered from soils has expanded beyond southeast Asia and northern Australia, and now includes large areas of the Middle East and South/Central America [reviewed in [14], [15]]. Concurrently, an increase in melioidosis cases has been observed in many of these regions. Diabetes is a major risk factor for acquiring these infections, as well as for developing severe disease. Because diabetes rates are rapidly increasing worldwide, it is likely that the number of melioidosis cases will also rise substantially. Together with the reports that *B. pseudomallei* possesses virulence attributes that make it attractive for misuse in bioterrorism-related releases has generated great interest in better understanding how this pathogen can so efficiently evade our immune defenses [97], [98]. Neutrophils have recently been reported to be prominently associated with *B. pseudomallei* infections in *vivo* and possess many qualities that would suggest they are capable of promoting host clearance [42]. However, the previous *in vitro* studies have reported disparate findings as to abilities of neutrophils to control these infections [55], [56], [57]. While our findings strongly indicate that neutrophils can efficiently clear *B. pseudomallei*, these activities appear to be dependent on the presence of critical C3 levels deposited on the surface of this pathogen. Thus, the differences observed between our findings and those of some previous publications may be partly attributed to differences in the serum concentration used, in the experimental set-up for neutrophil analyses, or even the handling of the bacteria and neutrophils [55], [56], [57]. However, based on the experimental methods described in these publications, there is no obvious indication for the discrepancies in the reported results. Our findings that neutrophil-generated NADPH-oxidase activity is important for *B. pseudomallei* clearance also strongly correlates with current *in vivo* studies reporting the significance of this pathway in melioidosis development [43], as well as the large number of recent reports associating the presence of neutrophil with improved outcome in *B. pseudomallei* infections [45], [55], [56], [57]. Several recent studies have also reported that neutrophils possess previously unappreciated abilities to interact directly and indirectly with other immune cells including macrophages, dendritic cells, NK cells, and T cells, and modulate their functions to further enhance disease resolution [reviewed in [99], [100]]; this suggests that neutrophils may possess additional important roles in resolution of melioidosis.

In conclusion, it is apparent that human neutrophils are able to effectively internalize and kill *B. pseudomallei* that have been opsonized with complement. The deposition of sufficient levels of complement components on the bacterial surface is critical for these processes. The mechanism of killing can largely be attributed to ROS production by these appropriately activated neutrophils. The primary predisposing condition for melioidosis, diabetes, is known to reduce chemotactic activity, phagocytosis, and microbicidal activities including decreased production of reactive oxygen species (reviewed in [44] and [45], [46]). Therefore, therapeutic strategies to promote recruitment of neutrophils to sites of infection and restore their phagocytic and bactericidal activities may allow diabetic individuals to control and limit the systemic spread of *B. pseudomallei* infections. Furthermore, modulation of neutrophil recruitment and function and/or complement deposition on the surface of *B. pseudomallei* may enhance the resolution of melioidosis regardless of predisposing condition.
